# Five-Year Comparison Results Between Clinically Severely Affected Tetralogy-of-Fallot Patients Initially Treated by Right Ventricular Outflow Stenting and Pink-Fallot Patients Undergoing Single-Step Correction

**DOI:** 10.3390/jcdd11120398

**Published:** 2024-12-11

**Authors:** Anton Alexandrovich Lyapin, Irina Nikolaevna Lyapina, Alexandra Alexandrovna Rumiantseva, Roman Sergeevich Tarasov

**Affiliations:** Laboratory of X-Ray Endovascular and Reconstructive Cardiovascular Surgery, Department of Cardiovascular Surgery, Federal State Budgetary Institution “Research Institute for Complex Issues of Cardiovascular Diseases”, Blvd. Named After Academician L.S. Barbarasha, 6, 650002 Kemerovo, Russia; lyapaa@kemcardio.ru (A.A.L.); anikaa@kemcardio.ru (A.A.R.); tarars@kemcardio.ru (R.S.T.)

**Keywords:** congenital heart disease, tetralogy of Fallot, palliative treatment, stenting of right ventricular outflow tract

## Abstract

The purpose: Evaluation of the short-term and long-term results of a phased correction of the tetralogy of Fallot (ToF) with stenting of the right ventricular outflow tract (RVOT) in comparison with a one-stage total correction (TC) of the defect. Materials and methods: Two groups of patients with classical ToF were formed. Group 1 (n = 25; median age = 72 days) was initially represented by children with ToF with a more severe clinical status (median weight = 3.6 kg, with more pronounced cyanosis and with comorbidities). The children of group 1 underwent the first stage of RVOT stenting and the second stage of TC of ToF. Group 2 (n = 25) was represented by older patients, with a higher body weight and SpO2 level, and they underwent a single-stage TC of the defect. Results: The application of a step-by-step ToF correction approach with RVOT stenting in low-weight newborns with severe hypoxemia demonstrated an equivalent effect on SpO2 dynamics—reverse remodeling of the heart—when compared with a less severe cohort of patients who underwent simultaneous TC of classical ToF. After RVOT stenting in children from group 1, the median SpO2 increased from 80% to 94.5%, the median Z value of the pulmonary artery trunk decreased from −3.46 mm to −2.54 mm, and the median index of end-diastolic volume of the left ventricle decreased from 23.07 mm/m^2^ to 57.6 mL/m^2^. TC of ToF in children from group 1 with a phased strategy of correction of the defect was no less successful than in children who underwent simultaneous TC. In the long-term follow-up period after TC of ToF, children from both groups, who were obviously unequal in their initial status, were practically comparable in clinical characteristics, exhibiting features of cardiac remodeling and achieving endpoints. And there were no significant differences between the two groups in the frequency of reaching the endpoints such as re-operations, cerebrovascular events, and death during the annual, three-year, and five-year follow-up period. Conclusions: The strategy of RVOT stenting followed by TC of ToF in a severe group of children demonstrated comparable results compared with the results of simultaneous TC of ToF in a more stable group of patients during the in-hospital, annual, three-year, and five-year follow-up periods.

## 1. Introduction

Tetralogy of Fallot (TF) is a severe multicomponent heart defect, which include right ventricular outflow tract (RVOT) stenosis, a ventricular septal defect (VSD), aortic dextroposition and right ventricular (RV) hypertrophy [1]. This disease is the most widespread cyanotic congenital heart defect (CHD) [2]. Due to severe RVOT stenosis, the blood in the child’s body bypasses the lungs and circulates through the organs and tissues without being saturated with oxygen and leads to a blue skin shade as a result. Half of the children with TF die before reaching the age of three without surgical correction.

RVOT stenting is a modern minimally invasive type of palliative correction specifically in children with a cyanotic form of TF. RVOT stenting was first described by Gibbs et al. in 1997. They performed this procedure in four patients with congenital heart disease, including the hypoplastic pulmonary artery (PA) [3]. RVOT stenting increases pulmonary blood flow after the birth of a child with TF. This palliative intervention has an inherent advantage compared to open ductus arteriosus stenting or modified Blalock Taussig shunt (MBTS) application, because RVOT stenting provides more physiological blood circulation. That is, the blood in the pulmonary artery comes from the venous system, not from the arterial one. As a result, there is a more efficient oxygen exchange and prevention of diastolic outflow, which occurs during the “physiology of bypass surgery”, as well as the absence of a decrease in perfusion pressure in the aorta and coronary arteries [4]. That significantly “changes the rules of the game” for symptomatic newborns with TF. RVOT stenting may be more beneficial than installing a stent in an open ductus arteriosus or applying an MBTS in young children with concomitant diseases predisposed to necrotizing enterocolitis, which may be aggravated by diastolic outflow. Also, RVOT stenting avoids the risks inherent in open surgery with systemic pulmonary bypass surgery.

But the question of the long-term results of this palliative correction remains not fully studied. It is believed that the effectiveness of this method is limited to only a very short period of time.

The objective of this work was to evaluate the short-term and long-term results of RVOT stenting as a step repair in comparison with one-stage TC of the defect.

## 2. Materials and Methods

This was a pilot study (registration number № 124041800039-2, kemcardio.ru) in patients with tetralogy of Fallot. There were two groups of patients in this study. To reduce the influence of preoperative factor differences on the study results, “pseudo-randomization” (propensity score matching) was carried out between groups using the “nearest neighbor” search method in a 1:1 ratio for the following 6 preoperative factors: age, gender, body weight, RVOT pressure gradient, the classic form of TF with RVOT stenosis diagnosis, and Z value of the PA trunk [5]. A total of 50 patients were included in the analysis after pseudo-randomization (group 1, RVOT stenting—n = 25; group 2, one-stage TC of TF—n = 25).

The prospective part of this study (group 1) included 25 newborns (12 boys and 13 girls) with the cyanotic form of classical TF and progressive two-stage correction of congenital heart disease (1st stage—stenting of the RVOT using bare metal stent PRO-Kinetic Energy (Biotronic, Lake Oswego, OR, USA); 2nd stage—TC of TF). The median age of children at the time of RVOT stenting was 72.0 [33.0; 111.0] days, the median weight was 3.6 [2.8; 4.4] kg, and the median arterial blood oxygen saturation (SpO2) was 80.0 [75.0; 83.5]%, respectively. There were more initially heavier newborn children in this group, and they had cyanosis, prenatal central nervous system damage, brain malformations and intrauterine growth retardation.

It was impossible to perform single-step TC in patients of group 1 because of severe hypoplasia of the pulmonary vascular bed. That is why all children from group 1 underwent stenting of the RVOT as a bridge to the TC. The median age in group 1 at the time of TC was 172.0 days.

Group 2 analysis was performed retrospectively. This group also included 25 newborns, 12 boys and 13 girls, with the classic form of TF, who underwent single-step TC of congenital heart disease. This group of patients was older, with a higher body weight and SpO2 level at the time of TF correction in comparison with the patients in group 1 (Table 1).

All patients from the both groups underwent standard transthoracic echocardiography. In addition to such standard indicators as the size and volume of the heart chambers, valve apparatus, ejection fraction (EF) of the left ventricle (LV), and the pulmonary artery pressure (PAP), the size of the PA trunk, the size of the PA branches, and the RVOT pressure gradient were also analyzed. The findings were used for a comprehensive assessment of the pulmonary vascular bed with the calculation of the z-score for the pulmonary artery and its branches. The LV end-diastolic volume index (LVED) was calculated using the following formula: LVED/child’s body surface area.

The primary endpoint of this study was a composite endpoint that included a combination of such adverse events as death from any cause and unplanned reoperation. Also, this study investigated such endpoints as cardiac conduction disorders requiring installation of a pacemaker, acute cerebrovascular accidents (ACVAs), and bleeding requiring resternotomy. We also assessed the safety of the RVOT stenting procedure using indicators such as death and bleeding caused by the procedure. We assessed the effectiveness of RVOT stenting with the gradient increase that required additional stent inflation or the application of an MBTS.

The follow-up included the examination of patients in a year, 3 years and 5 years after TC of TF in two groups.

The research results were processed using the application package Statistica for Windows 10.0 (StatSoft Inc., Tulsa, OK, USA). Initially, the data distribution was checked for normality of distribution using the Kolmogorov–Smirnov test and, due to the lack of normal data distribution, nonparametric tests were used. Quantitative indicators are presented as the median and interquartile range (25th and 75th percentile). Nonparametric Mann–Whitney tests were used for assessing differences in quantitative indicators, and the Wilcoxon test was used for assessing dynamics in one group. Qualitative indicators are represented by the frequency distribution of the characteristic. To assess differences in qualitative indicators, the Pearson Chi-square test was used. The critical level of statistical significance was taken to be 0.05.

## 3. Results

The early postoperative period in 24 children passed without significant features after RVOT stenting (group 1), which allowed to prepare these children for TC of TF. One child in group 1 experienced hypoxemic syndrome and dyspnea-cyanotic attacks became more frequent 2 months after stenting. The child was sent to the operating room and a systemic-pulmonary shunt was formed as an emergency operation. The patient was extubated after admission to the intensive care unit. Clinical signs of respiratory failure (RF) developed 2 h after extubation, and there were symptoms of desaturation, metabolic acidosis, bradycardia, and hypotension. Resuscitation efforts were unsuccessful and biological death was declared.

There was no bleeding requiring sternotomy, as well as no vascular access complications, tricuspid valve affection, and arrhythmias including heart block, and we did not observe mortality in cases of RVOT stenting. There was also no need to take children back to the cath lab to inflate the stent. Based on this, we can conclude that RVOT stenting is an effective and safe method of palliative care for cyanotic TF.

Children in group 1 had significantly gained weight to a median of 5.5 kg, and the severity of cyanosis had significantly decreased with an increase in SpO2 to a median of 94.5% by the time of TC (Table 2).

One of the main findings of this study was the body weight of patients from the RVOT stenting group (Group 1): it was comparable with body weight of children who underwent single-step TC (Group 2) at the time of hospitalization for TF TC. There were no significant differences in age, arterial blood saturation and RVOT pressure gradient. RVOT stenting helped to prepare an initially more severe group of patients for TF total correction. At the same time, the significant differences in LVED index were identified due to the more severe initial status of patients in the RVOT stenting group (Table 3).

Children from two groups underwent TC of the defect. Intraoperatively, the VSD was closed with a xenopericardial patch, after which the stenosis of the RVOT was eliminated by excision of abnormal septal trabeculae, and, if necessary, transannular repair of the RVOT was also performed.

Comparing the perioperative features of TC of TF in children from group 1, the median time of cardiopulmonary bypass (CPB) was 127.5 [119.0; 144.5] minutes and the aortic clamping time was 86.0 [77.5; 94.7] minutes; therefore, the CPB time in children from group 2 was 110.0 [93.0; 119.0] minutes and the aortic clamping time was 70.0 [66.0; 79.0] minutes. This is explained by additional manipulations to explant the stent from the RVOT in addition to the main stages of the TC.

In relation to outcomes, there was no significant difference in the time of mechanical ventilation. Postoperative rehabilitation time in the intensive care unit and cardiac surgery department, SpO2 level, and RVOT pressure gradient also did not differ significantly. This indicates a positive effect of palliative repair in the RVOT stenting case by preparing the pulmonary-circulation-normalizing antegrade blood flow in the PA system. This is also evidenced by the fact that children from group 1 actively gained weight between RVOT stenting and the TC period, which also had a beneficial effect on the outcomes of TC.

### 3.1. Early Postoperative Period (After TF TC)

#### 3.1.1. Group 1, RVOT Stenting

In the early postoperative period, within two days after correction, 20.8% of children (n = 5) from group 1 had hypervolemia of the pulmonary circulation, which did not allow to stop mechanical ventilation and start spontaneous breathing. There was a respiratory failure (RF) development in an attempt to extubate the trachea, and a large amount of pink sputum was sanitized in the endotracheal tube, which indicated the presence of major aortopulmonary collateral arteries (MAPCAs). The children were sent to the cath lab, where the MAPCAs were verified and successful occlusion was performed.

In 8.3% of children (n = 2), severe RF was also observed the second day after TC due to paresis of the cupula of the diaphragm. It required its plication to achieve a positive clinical effect. In the RVOT stenting group, 4.2% of children (n = 1) developed complete atrioventricular block the fifth day after TC, which required the pacemaker implantation. One child had an increase in the pressure gradient in the pulmonary artery branches up to 54 mm Hg in 2 months after TC. This patient was routinely hospitalized for PA plastic surgery under CPB conditions. Operation was successfully completed.

A proportion of 8.3% (n = 2) of deaths were observed in the early postoperative period in this group of patients. The first child had cardiac arrest the next morning after the correction, direct cardiac massage was performed, and extracorporeal membrane oxygenation (ECMO) was connected. Due to intraventricular bleeding, ECMO was stopped in seven days and biological death was declared.

The second child underwent successful TC, but after discharge from the hospital, the child died of non-cardiac causes due to multiple congenital malformations.

After discharge, contact with the relatives of one child who underwent TC was lost; the fate of the patient is unknown.

#### 3.1.2. Group 2

In group 2 (patients with single-step TF TC), one patient had an increased rate of blood flow through the drains in the first postoperative hours, which became the reason for revision and resternotomy. The source of bleeding was the arterial branch of the periosteum of the sternum. The exploration was successful.

In 10 days after surgery, a child from group 2 had an ischemic ACVA in the middle cerebral artery, which led to left-sided hemiparesis and convulsive syndrome. One child also had permanent atrioventricular block and pacemaker implantation was performed.

One child (4%) had bradycardia with the transition to asystole, resuscitation measures were carried out in full without effect, and biological death was declared one day after surgery.

Pulmonary circulation hypervolemia was observed in the early postoperative period in 8% of children (n = 2), MAPCAs were verified, and endovascular occlusion was successfully performed. Also, 8% of children (n = 2) had severe RF, which was a consequence of paresis of the cupula of the diaphragm. These children were admitted to the operating room for plication of the cupula of the diaphragm with a positive clinical effect (Table 4 and Table 5).

Children from two groups were routinely admitted to the cardiac center for screening examinations in a year after the TC. A proportion of 62.5% of patients (n = 15) from group 1 did not come to the face-to-face visit one year later. A proportion of 12.5% (n = 3) of children did not come, because they did not reach the required postoperative observation period, and 8.3% (n = 2) of the children moved to another region and so it was impossible to appear at the clinic. All patients were contacted by telephone to evaluate some endpoints. A proportion of 92% (n = 23) of children from group 2 came to the clinic for examination, whereas 4% (n = 1) did not reach the required postoperative observation period. Each of those who appeared underwent transthoracic echocardiography according to a standard protocol. According to the electrocardiogram (ECG), all children maintained sinus rhythm, excluding the child who had a pacemaker. SpO2 was not assessed, due to the absence of recurrence of cyanotic syndrome. There were no hospitalizations due to deterioration of the condition and no reoperations.

### 3.2. Postoperative Period—Monitoring at 3 Years After the Repair

After 3 years, 45.8% (n = 11) of children from the RVOT stenting group continued follow-up, whereas 25% (n = 6) did not reach the required follow-up period. One child from this group had a gradient increase in the pulmonary artery according to echocardiography, which required hospitalization and reoperation in the cardiopulmonary bypass condition to repair the ostial stenosis and left pulmonary artery plastic with a xenopericardial patch.

A proportion of 60% (n = 15) of children from the group of single-step TC came for a routine examination, whereas 36% (n = 9) did not reach the required monitoring period. According to ECG data, sinus rhythm was present in all patients. There were no reoperations in this group.

### 3.3. Postoperative Period Monitoring at 5 Years After the Repair

A proportion of 33.3% (n = 8) of children in the RVOT stenting group had a 5-year follow-up period, whereas 37.5% (n = 9) did not reach the required follow-up period. A proportion of 20% (n = 5) of children from the group of single-step TC had a 5-year follow-up period, whereas 76% (n = 19) did not reach the required follow-up period. There were no hospitalizations due to deterioration of the condition and no reoperations.

## 4. Discussion

Currently, there are different approaches to the management of patients with TF. It is recommended to perform single-step TC in case the disease is favorable without cyanosis, with normal body weight, and without severe comorbidity. But in clinical practice, specialists in congenital heart disease very often encounter a severe cohort of patients for whom it is impossible to perform single-step TC, so they have to resort to a palliative strategy. However, the superiority of a palliative approach in comparison with total correction of TF in newborns is ambiguously demonstrated in studies [6]. As part of palliative care, open heart surgery with the formation of a systemic-pulmonary anastomosis (MBTS) is more often performed before TC of TF. Despite its widespread use, MBTS also has its drawbacks: uneven development of the pulmonary artery, pulmonary artery stenosis at the anastomosis location, shunt occlusion, and hemodynamic instability in the early postoperative period [7]. At the same time, such a palliative method as RVOT stenting also increases pulmonary blood flow after the birth of a child with TF and has the main advantage of being minimally invasive. However, the results of using this method are still contradictory [8].

Analyzing the literature, we did not find any works devoted to a comparative analysis of patient outcomes during single-step TC of TF and palliative correction of the defect with initial stenting of the RVOT and subsequent TC.

In this study, we compared two groups of children, one of which followed the palliative repair of TF with RVOT stenting (group 1), and the second one underwent single-step total correction of TF (group 2). The choice of a staged strategy in the children of group 1 was chosen due to their more severe clinical status, comorbidity, severe cyanosis, and low weight. RVOT stenting improves pulsatile venous flow to the PA, improving SpO2 without reducing aortic diastolic pressure and, as a consequence, without reducing coronary perfusion. In our study, in the RVOT stenting group, after the palliative stage and before the children were hospitalized for TC, there was a significant increase in weight with a median increase of 1.9 kg, an improvement in SpO2 immediately after installation of the stent in the RVOT from 80% to 94.5%, an increase in the median LVED index by 9.23 mL/m^2^, and a positive effect on the development of the pulmonary circulation. The study by Ghaderian M. et al. also demonstrated that stenting the RVOT helps reduce hypoplasia of the pulmonary circulation vessels. Comparing RVOT stenting with MBTS, the authors conclude that these two techniques are comparable [9]. According to Quandt D, a shorter intensive-care duration and hospitalization period characterized the group with a stented RVOT; however, the frequency of re-stenting in these patients was higher [4]. In our study, the duration of intensive therapy and the time spent in the clinic were longer in the RVOT stenting group compared to the TC group. This is explained by the fact that group 1 had an obviously more severe clinical status, which determines such results. Bentam J. et al. found better survival after RVOT stenting (odds ratio, 0.25; 95% confidence interval, 0.07–0.85) compared with MBTS [10], whereas Glatz et al. found no difference in survival (odds ratio, 0.64; 95% confidence interval, 0.28–1.47) [11].

The study of Abumehdi M. included 26 children who underwent RVOT stenting, 15% of children died (n = 4) [12]. In our study, the mortality rate was 16%. This is due to the fact that in the Russian Federation, RVOT stenting is not a method of choice and this technique is not included in the national recommendations for the management of children with TF [13]. We performed this intervention off-label for a severe cohort of children, 40% of whom were premature and other 48% had perinatal damage of the central nervous system, multiple congenital malformations, including brain and intrauterine development delays.

In this work, the use of an approach of palliative correction of TF with RVOT stenting in low-birth-weight newborns with severe hypoxemia demonstrated an equivalent effect on SpO2 dynamics and reverse cardiac remodeling in comparison with a less severe cohort of patients who underwent single-step TC of classical TF. The basis for obtaining such an encouraging positive clinical effect is the satisfactory result of implanted coronary stents in the outflow tract biocompatibility, which we previously studied in the research with a histological assessment of explanted stents [14]. Thus, such palliative correction as RVOT stenting helped to prepare a severe cohort of children for TC. At the time of admission to the clinic for TC, the initially unequal groups of children in their clinical status were almost equal in their indicators after RVOT stenting, and TC in this group was no less successful than in children who underwent single-step TC.

In the long-term follow-up period, children from two groups obviously unequal in terms of initial status were practically comparable in clinical characteristics, features of cardiac remodeling and endpoints. Assessing long-term results, we focused on such endpoints as death from all causes, repeated reoperation for the main disease, cardiac conduction disorders requiring pacemaker implantation, and ACVA. There were also no significant differences between the two groups in one-year, three-year, and five-year follow-up periods.

## 5. Summary

The strategy of RVOT stenting before TC in a severe group of children with TF demonstrated comparable results in comparison with the results of single-step TC in a more stable group of patients during the in-hospital, one-year, three-year, and five-year follow-up periods.

## Figures and Tables

**Table 1:** Comparison of initial clinical and demographic characteristics and transthoracic echocardiography parameters in patients with planned staged and single-stage repair of TF.

Parameters	RVOT Stenting (Group 1; n = 25)	Total Repair (Group 2; n = 25)	*p*-Value
Clinical and Demographic Characteristics			
Age (days)	72.0 [33.0; 111.0] at the time of RVOT stenting	121.5 [63.7; 153.0] at the time of TC	0.0182
Age at the time of TC (days)	172.0 [118.0; 247.5]	121.5 [63.7; 153.0]	0.02
Weight, kg	3.6 [2.8; 4.4]	5.2 [4.7; 5.6]	0.00002
SpO2 before repair, %	80.0 [75.0; 83.5]	94.0 [92.0; 95.5]	0.000001
Transthoracic Echoradiography Parameters			
Pulmonary trunk (mm)	5 [4; 6.1]	8.5 [6; 9.6]	0.00007
LVED index (mL/m^2^)	23.07 [17.24; 29.41]	57.6 [40.5; 67.1]	0.0000001
PA trunk Z value (mm)	−3.46 [−4.25; −1.98]	−0.37 [−2.99; 0.35]	0.0008
Right PA Z value (mm)	−0.76 [−1.78; 0.39]	0.855 [0.012; 1.71]	0.002
Left PA Z value (mm)	0.015 [−1.53; 0.66]	1.05 [−0.095; 2.19]	0.006
RVOT gradient pressure, mm Hg	72.0 [60.0; 77.0]	72.0 [65.5; 80.0]	0.429
Index Nakata, mm^2^/m^2^	137.3 [91.5; 177.8]	208.1 [175.9; 293.8]	0.003
Index McGoona	0.8 [0.6; 0.8]	0.9 [0.9; 1]	0.0001

**Table 2:** Dynamics of the clinical and functional status of patients in group 1 (RVOT stenting) from the moment of hospitalization for palliative repair to TF total correction.

Parameters	Before RVOT Stenting (n = 25)	Before TF TC (n = 24)	*p*-Value
Weight, kg	3.6 [2.8; 4.4]	5.5 [4.8; 6.2]	0.00004
SpO2, %	80.0 [75.0; 83.5]	94.5 [91.7; 98.0]	0.000035
PA trunk (mm)	5 [4; 6,1]	7 [5; 8]	0.1005
LVED index (mL/m^2^)	23.07 [17.24; 29.41]	32.3 [25.9; 50]	0.000061
PA trunk Z value (mm)	−3.46 [−4.25; −1.98]	−2.54 [−3.56; −1.25]	0.079
Right PA Z value (mm)	−0.76 [−0.27; 0.38]	0.85 [0.01; 1.71]	0.057
Left PA Z value (mm)	−0.19 [−1.08; 0.44]	1.05 [−0.09; 2.19]	0.58
RVOT gradient pressure, mm Hg	72.0 [60.0; 77.0]	70.0 [66.0; 90.0]	0.1712
Index Nakata, mm^2^/m^2^	137.3 [91.5; 177.8]	137.7 [124.1; 172.9]	0.426
Index McGoona	0.8 [0.6; 0.8]	0.7 [0.6; 0.8]	0.35

**Table 3:** Comparison of the two groups at the time of hospitalization for radical correction of TF.

Parameters	Group 1 RVOT Stenting Patients Before TC (n = 24)	Group 2 Before Single-Step TC (n = 25)	*p*-Value
Age at the TC time, days	172.0 [118.0; 247.5]	121.5 [63.8; 153.0]	0.002
Weight, kg	5.5 [4.8; 6.3]	5.2 [4.7; 5.6]	0.1009
SpO2, %	94.5 [917; 98]	94.0 [92.0; 95.5]	0.315
PA trunk (mm)	7 [5; 8]	8.5 [6; 9.6]	0.106
LVED index (mL/m^2^)	32.3 [25.9; 50]	57.6 [40.5; 67.1]	0.003
PA trunk Z value (mm)	−2.54 [−3.56; −1.25]	−0.37 [−2.99; 0.35]	0.043
Right PA Z value (mm)	−0.07 [−0.27; 0.38]	0.85 [−0.01; 1.71]	0.022
Left PA Z value (mm)	−0.19 [−1.08; 0.44]	1.05 [−0.09; 2.19]	0.013
RVOT gradient pressure, mm Hg	70.0 [66.0; 90.0]	72.0 [65.5; 80]	0.9
Index Nakata, mm^2^/m^2^	137.7 [124.1; 172.9]	208.1 [175.9; 293.8]	0.004
Index McGoona	0.7 [0.6; 0.8]	0.9 [0.9; 1]	0.9

**Table 4:** Hospital outcomes at the time of discharge after TC including death from all reasons and unplanned reoperations.

Parameters	RVOT Stenting, Group 1 (n = 23)	Single-Step, TC Group 2 (n = 24)	*p*-Value
Hospital Outcomes			
CPB, days	2.5 [1.0; 4.0]	2.5 [1.0; 5.0]	0.961
Intensive care unit period, days	7.5 [5.0; 10.2]	5.5 [4.0; 10.5]	0.335
Cardiac surgery department, days	16.5 [12.5; 27.25]	13.5 [12; 16.25]	0.487
Surgery Efficacy Indicators			
SpO2, %	98.0 [98.0; 99.0]	98.0 [98.0; 99.2]	0.353
RVOT gradient pressure, mm Hg	16.5 [9.0; 26.0]	12.0 [10.0; 20.0]	0.754
LVED index (mL/m^2^)	56.52 [46.8; 68.8]	67.81 [59.81; 75.89]	
Clinical Endpoints			
Combined endpoint (death + unplanned operation)	0	0	
Bleeding with resternotomy	0	1	
ACVA	0	1	
Death	3	1	
Pacemaker implantation	1	1	
Paresis of the diaphragm cupula	2	2	

**Table 5:** Postoperative period—monitoring at 1 year, 3 years, and 5 years after TC.

RVOT Stenting, Group 1			
Reoperation	1-Year Monitoring (n = 15)	3-Year Monitoring (n = 11)	5-Year Monitoring (n = 8)
Age (months)	22 [17; 23]	42 [38.25; 46.75]	66 [64.25; 73.0]
Weight	8 [7.1; 8,75]	10 [9.1; 12.7]	16 [14.75; 16.33]
LV EF	74 [70; 76]	69 [68.5; 74.5]	66 [66; 72.75]
LVED index (mL/m^2^)	71.42 [57.17; 72.17]	64.06 [51.66; 68.68]	100 [55.34; 102.7]
PA gradient	12 [9.5; 25.5]	13 [11.5; 18.75]	19 [14.25; 19.25]
Death	0	0	0
Reoperation	0	1	0
**Single-Step TC, Group 2**			
**Reoperation**	**1-Year Monitoring (n = 23)**	**3-Year Monitoring (n = 15)**	**5-Year Monitoring (n = 7)**
Age (months)	15.5 [14.7; 18]	40 [39; 47]	66 [64; 66.5]
Weight	8.5 [7.5; 9.4]	13.5 [10.5; 14.35]	17 [15.75; 18.35]
LV EF	71 [67; 74]	69 [67; 73]	70 [68; 71.5]
LVED index (mL/m^2^)	63.82 [42.25; 86.4]	69.76 [63.45; 81.54]	120.51 [97.82; 150.87]
PA gradient	13.5 [10.2; 19.5]	11 [9.5; 14]	11 [8; 15]
Death	0	0	0
Reoperation	0	0	0

## Data Availability

The database of the study https://cloud.mail.ru/public/CShs/M3skUiNHD (accessed on 1 December 2024).
